# Predictors of outcome in phaeochromocytomas and paragangliomas

**DOI:** 10.12688/f1000research.12419.1

**Published:** 2017-12-21

**Authors:** Marlo Nicolas, Patricia Dahia

**Affiliations:** 1 University of Texas (UT) Health Cancer Center, San Antonio, TX, USA; 2 Department of Pathology, San Antonio, TX, USA; 3 Division of Hematology and Medical Oncology, Department of Medicine, UT Health San Antonio, TX, 78229, USA

**Keywords:** pheochromocytoma, paraganglioma, risk factor, prognostic prediction

## Abstract

Phaeochromocytomas and paragangliomas (PPGLs) are catecholamine-secreting neuroendocrine tumours characterised by high rates of heritability and genetic heterogeneity. Despite advances in the genetic diagnosis and improved understanding of the molecular aberrations underlying these tumours, predictive markers of malignancy remain scarce, limiting the outlook of patients with metastatic PPGL. The identification of robust predictive markers remains the most pressing challenge in PPGL management, so that the potential of targeted therapy to impact patient care can be fully realised.

## Overview of phaeochromocytomas and paragangliomas

Phaeochromocytomas and paragangliomas (PPGLs) are catecholamine-secreting tumours of neural crest origin, which arise in the adrenal medulla and extra-adrenal paraganglia, respectively ^1^. Despite advances in the field, risk factors of PPGL malignancy remain limited and few options are available for patients with metastatic PPGL ^2^.

Over the past two decades, progress in the molecular genetics of PPGLs has provided key insights into the primary driver event underlying their pathogenesis. PPGLs are genetically heterogeneous tumours caused by mutations in over 20 distinct genes ^3^. A pathogenic mutation, either germline or somatic, can be identified in two-thirds of the tumours largely in a mutually exclusive manner ^4^. These various aberrations have led to a classification of PPGLs into distinct biological subgroups, as illustrated in Table 1. However, these subgroups have poor prognostic discriminatory power.

**Table 1.** Molecular classification of phaeochromocytomas and paragangliomas.

Subtype	Mutated gene	Mutated cell	Risk of malignant phaeochromocytomas and paragangliomas
Kinase signalling	*RET*	Germline or somatic	L
	*TMEM127*	Germline	L
	*NF1*	Germline or somatic	L/M
	*HRAS*	Somatic	L
	*FGFR1*	Somatic	?
	*H3F3A*	Mosaic	?
	*MAX*	Germline or somatic	L/M?
Pseudohypoxia	*VHL*	Germline or somatic	L
	*SDHA*	Germline	L
	*SDHB*	Germline (rare somatic)	H
	*SDHC*	Germline	L/M
	*SDHD*	Germline	L
	*SDHAF2*	Germline	L
	*EPAS1*	Mosaic or somatic	L/M
	*FH*	Germline	L/M?
	*MDH2*	Germline	?
	*EGLN1*	Germline	?
Wnt signalling	*MAML3*	Somatic (fusion)	H?
	*CSDE1*	Somatic	?

?, insufficient or recent data or both; H, high; L, low; M, moderate.

Most PPGLs have a benign course and can be successfully treated by surgery. Approximately 15% of PPGLs are malignant and not curable by current therapies ^2^. In fact, malignancy status in PPGLs is predicated on the detection of metastases at distant sites unrelated to the paraganglial tissue. Therefore, by definition, malignant PPGLs are diagnosed late. A second category of PPGLs, though not fulfilling these metastatic criteria, comprises tumours with aggressive clinical behaviour that involves fast growth, multiple recurrences, or both, some of which are inoperable, thus leading to poorly controlled disease. As a group, ‘aggressive’ and metastatic PPGLs represent a substantial number of patients for whom therapeutic options remain scarce. Currently, predictive factors for malignant PPGL and for ‘aggressive’, non-metastatic PPGL are limited and imprecise ^5^. An intrinsic limitation of the current definition of poor outcome is the inability to distinguish truly ‘benign’ PPGLs from those that eventually will progress to malignant or aggressive disease in the absence of long-term clinical follow-up. This shortcoming reduces the specificity of predictive models. Therefore, there is a clear need for markers that allow precise and early recognition of tumours with negative clinical outcomes.

We posit that despite the indisputable value of molecular discoveries to the genetic diagnosis of PPGLs, this progress has yet to be translated into broad, reliable prognostic predictions. Novel clinical trials targeting specific molecular aberrations found in PPGLs are beginning to emerge and are expected to expand the therapeutic options available for patients with metastatic disease ^2^. The prospect of more effective treatment of advanced malignant PPGL is encouraging, yet the identification of predictive markers with the potential to impact patient care remains the most pressing challenge in PPGL management.

## Advances in phaeochromocytoma and paraganglioma biology

PPGLs are infrequent neuroendocrine tumours that arise from cells of the autonomic lineage, sympathetic or parasympathetic, derived from the neural crest ^1, 6^. Sympathetic lineage cells, also known as chromaffin cells, give rise to the more frequent form of tumour presentation (that is, PPGLs that have the potential to secrete catecholamines), whereas parasympathetic-derived paragangliomas are histologically related but do not usually have catecholamine-secreting ability. PPGLs have the highest rate of heritability of all human tumours; approximately 40% are caused by an inherited autosomal dominant mutation in one of more than a dozen susceptibility genes ^1^ ( Table 1). Thus, the identification of an inherited pathogenic PPGL mutation has immediate clinical impact, as it can lead to early diagnosis of at-risk individuals in families through genetic testing. In addition, since hereditary PPGLs often present as part of multi-tumour syndromes, mutation detection can also lead to planned surveillance and early diagnosis of co-occurring cancers in probands and in their mutation-carrier relatives ^1^.

PPGLs are often benign and curable by surgery. However, these tumours can be clinically aggressive with multiple recurrences or can spread to non-chromaffin derived, metastatic sites, including liver, lung, bone, and lymph node ^2, 7^. Both hereditary and sporadic PPGLs can progress to metastatic disease. The overall survival rate of patients with metastatic PPGL is 60% in five years, although there is extensive heterogeneity ^2, 8^. It has been recognised that a substantial number of patients with untreated malignant PPGL—almost half by some accounts—can remain stable without disease progression for months after the initial diagnosis ^8, 9^. However, currently, it is not possible to anticipate, prior to treatment, the growth and spread rate of individual metastatic PPGLs. The ability to distinguish more indolent tumours from fast-growing, highly symptomatic cases would have obvious impact on clinical decision-making. For example, patients with slow-growing, oligo/asymptomatic tumours could potentially benefit from active surveillance before initiating any therapy, as recently advocated for low-risk cancers as a way to reduce negative consequences of overtreatment ^10^.

On the other end of the spectrum, PPGLs that do not metastasize but are highly recurrent, either locally or at new paraganglial sites, can pose a therapeutic challenge from the surgical perspective, either because of tumour location or multiple prior interventions that limit surgical success or because of patients’ catecholamine-related symptoms ^11^. These ‘aggressive non-metastatic’ tumours share some of the risk factors associated with metastatic PPGLs ^5^. Recognition of precise risk factors for recurrence could provide the opportunity for more tailored therapeutic planning and follow-up.

## Clinical, anatomical, and histological risk factors in phaeochromocytomas and paragangliomas

Distinguishing benign PPGLs from tumours with metastatic potential is not possible on histologic grounds. Conventional approaches to estimate proliferative potential in cancers, such as the well-known Ki-67 index, measured by immunohistochemistry, has proven to be a reliable indicator of proliferating paraganglioma/phaeochromocytoma cells and for predicting tumour progression, when positive ^12^. However, the Ki-67 labelling index is often low or negative in PPGLs, limiting its use as a single prognostic indicator ^13^. In view of this limitation, a scoring system involving multiple histological features, known as PASS (phaeochromocytoma of the adrenal gland scaled score), was developed in an attempt to identify phaeochromocytomas (but not paragangliomas) with metastatic potential ^14^. Subsequent studies applying the PASS revealed significant variation in the interpretation of the different components, limiting its clinical utility ^15^. A second scoring system, GAPP (grading system for PPGL), which also included paragangliomas, was subsequently developed ^16^. Acknowledging the limitations of histomorphology alone in predicting PPGL biologic behaviour, GAPP includes, in addition to morphological parameters, the type of catecholamine secreted by the tumours. This feature was meant to function as a surrogate for tumour location, based on the long-established observation that norepinephrine (noradrenaline)-secreting tumours, which occur predominantly in paragangliomas, are more frequently malignant than those that secrete epinephrine (adrenaline), which are located almost exclusively in the adrenal gland (phaeochromocytomas) ^17^. However, despite this important addition, GAPP still has limitations ^18^. Catecholamine metabolites, metanephrines, are more faithful markers of tumour secretion than direct measurement of short-lived catecholamines. In addition, other relevant features which have been independently validated as prognostic markers, further discussed below, are not included in the score. Moreover, GAPP excludes paediatric patients, a group in whom the proportion of metastatic cases is twofold to threefold higher when compared with adults ^19^. It is possible that future iterations of this system will incorporate some of these parameters.

Non-histological predictors of PPGL malignancy, tumour size and location, have been recognised ^20^ ( Table 2). Tumours larger than 5 cm are more likely to spread, either because of an intrinsic accelerated growth rate or as a result of delayed diagnosis due to fewer symptoms (for example, as in the case of oligo- or non-secreting, less-differentiated tumours). Paragangliomas metastasize more often than phaeochromocytomas, regardless of the primary tumour size; as much as 20% of paragangliomas smaller than 5 cm are accompanied by metastases ^2, 8^. These two parameters have been incorporated into a new staging classification of PPGLs, based on the TNM (tumour, lymph node, and metastasis), which was recently developed by the American Joint Committee on Cancer. This classification was developed to help improve treatment planning of metastatic PPGLs. In its initial version, the TNM classification did not include molecular data ^2^.

**Table 2.** Parameters associated with poor outcome in phaeochromocytomas and paragangliomas.

Category	Parameter	Strength	Validation status ^a^
Molecular	Germline *SDHB* mutation	H	Y
Anatomical/Histological	Primary tumour size (>5 cm)	H	Y
Anatomical/Histological	Primary tumour location	H	Y
Anatomical/Histological	Proliferation index (Ki-67)	M (H when elevated)	N
Anatomical/Histological	Vascular invasion	L	N
Biochemical	Norepinephrine (noradrenaline) secretion	M	N
Biochemical	3-methoxytyramine levels (plasma)	M/H	?
Anatomical/Histological	c-Erb2 expression by immunohistochemistry	M/H	?
Molecular	Hypermethylation subtype	Interdependent with *SDHB* mutation?	?
Molecular	Pseudohypoxia subtype	Interdependent with *SDHB* mutation?	?
Molecular	Somatic *ATRX* mutation	?	?
Molecular	Other genetic mutations ( *MAX, FH*)	?	?
Molecular	Somatic MAML3 fusion	?	?
Molecular	WNT signalling subtype	Interdependent with *MAML3* fusion?	?
Molecular	RDBP hypermethylation	?	?
Molecular	Somatic SETD2 mutation	?	?

^a^By multiple independent and concordant sources of verification. ?, unknown/awaits further testing; H, high; L, low; M, moderate; N, no; Y, yes.

Plasma levels of 3-methoxytyramine, a sensitive indicator of tumour dopamine secretion, which can reflect poor differentiation of the catecholamine synthetic process by metastatic PPGLs, have shown initial promise as an additional biomarker, especially when combined with tumour size and location to predict the likelihood of malignancy in PPGLs ^20^. Importantly, high plasma 3-methoxytyramine was also detected in non-SDHB-related metastatic PPGLs, suggesting that this marker may have broader value, regardless of the genetic background of tumours ^20^. Prospective studies should provide further validation of the predictive power of 3-methoxytyramine in various clinical contexts.

## Molecular risk factors in phaeochromocytomas and paragangliomas

The best-known genetic predictor of malignancy is the presence of a germline mutation of the succinate dehydrogenase subunit B gene, *SDHB* ^21^ ( Table 2). SDH is a multi-unit enzymatic complex that is a component of both the Krebs cycle and the mitochondrial electron transport chain. Most patients with a germline *SDHB* mutation have extra-adrenal disease and approximately 50% of these patients progress to metastatic disease ^22^. In addition, patients with *SDHB* mutant malignant PPGLs have shorter median overall survival than non- *SDHB* mutant metastatic PPGLs (42 versus 244 months after the diagnosis of the first metastasis, respectively) ^21^. Mutations in other SDH component genes— *SDHA*, *SDHC*, *SDHD*, and *SDHAF2*—also lead to paragangliomas or phaeochromocytomas or both; however, malignancy is rarely associated with these tumours. *SDH* mutations cause a metabolic imbalance that leads to a DNA and histone hypermethylation phenotype; genes targeted by aberrant methylation are thought to be required for tumour development in these models ^23^. However, why mutations in *SDHB*, but not in the other *SDH* genes, specifically confer increased risk of malignancy is not known. Notably, despite its unquestionable role as an independent risk factor for malignancy, two-thirds of metastatic PPGLs do not have *SDHB* mutations, implying the existence of other markers of poor prognosis ^24^. Malignancy has also been reported in other genetic models of PPGLs but at much lower rates than those of SDHB mutant cases ^25, 26^.

A recent integrated analysis of PPGLs as part of The Cancer Genome Atlas (TCGA) using genomic, mRNA, and microRNA expression, methylation profiling, and protein expression arrays identified new pathogenic lesions in PPGLs ^4^. This study delineated four PPGL subtypes and identified nine outcome markers, as defined broadly by the time until the occurrence of distant metastases, local recurrence, or positive regional lymph nodes. Those included *SDHB* mutation, inclusion in the hypermethylation subtype, and inclusion in the pseudohypoxia subtype; the latter two are the groups that contain *SDHB* mutant tumours. Detection of a larger number of somatic mutations was also associated with worse outcome, although other studies did not observe such a correlation ^27, 28^. Importantly, novel molecular markers were found: *MAML3* (mastermind-like protein 3) fusions, *SETD2* (SET domain containing 2) or *ATRX* somatic mutations, and WNT-related expression subtype, which comprise the tumours with *MAML3* fusion tumours ^4^. Interestingly, almost all tumours carrying this fusion were phaeochromocytomas, in contrast with the predominant paraganglioma location of *SDHB* mutants, and represented a biological group distinct from the latter. Analysis including only the classic definition of malignant PPGLs (distant metastases) did not reveal any additional outcome markers. Future studies to validate these promising new candidate risk markers will be required.

Genes targeted by methylation that may associate with metastatic tumours, including RBDP, have also been identified and will need to be independently verified ^29^.

A recent study used multivariate logistic regression analysis and decision curve analysis of training and validation PPGL sets to develop a nomogram based on the combination of clinical (tumour size, location, and vascular invasion) and non-clinical (SDHB status and c-Erb2 expression) markers ^30^. The refined nomogram achieved good predictive power to distinguish PPGLs with metastatic potential. Independent verification of these findings in other cohorts is warranted. Importantly, this model lends itself to the addition of other recently identified markers and thus can prove adaptable to new discoveries.

## Prospects for prognostic prediction in phaeochromocytomas and paragangliomas

Fundamental to the successful identification of risk factors for malignant/aggressive PPGLs is a better understanding of their biological basis. For example, it is not clear whether a given PPGL is intrinsically aggressive at its onset or whether malignant transformation occurs later during tumour progression. The fact that an initiating driver mutation, *SDHB*, confers an independent risk of malignancy argues in favour of the former. However, as not all patients with an *SDHB* mutation develop metastatic disease, even within the same family (that is, carriers of the same mutation), other risk factors, either inherited through different alleles or acquired, might exist ^21^. Furthermore, metastatic PPGLs in children are more frequent than in adults independently of the heredity status, suggesting that tumours that manifest in childhood may be intrinsically more aggressive regardless of their genetic background ^19^. Additionally, the association between malignant PPGLs with structural abnormalities that might indicate genomic instability remains controversial ^4, 27, 28^. These unresolved questions expose our limited knowledge of the underlying biology of a malignant PPGL and represent an enduring obstacle to our ability to recognise these tumours at an early stage.

In conclusion, existing prognostic markers cannot fully predict PPGL behaviour. Recently found, and eventually future, candidate markers will require further verification and independent validation before they can be incorporated into outcome prediction algorithms. Prospective large multicentre studies that collect uniform, detailed long-term clinical follow-up data from heterogeneous population groups of patients and multidimensional large-scale data (comprehensive genetic, genomic, epigenetic, metabolic, immune, and proteomic) will be required to fill this important gap in our understanding of PPGLs and how that might impact patient care.

## Abbreviations

GAPP, grading system for phaeochromocytoma and paraganglioma; MAML3, mastermind-like protein 3; PASS, phaeochromocytoma of the adrenal gland scaled score; PPGL, phaeochromocytoma and paraganglioma; RBDP, negative elongation factor complex member E; *SDHB*, succinate dehydrogenase subunit B; TNM, tumour, lymph node, and metastasis.
